# Training community health workers for the COVID-19 response, India

**DOI:** 10.2471/BLT.21.286902

**Published:** 2021-11-25

**Authors:** Sachin S Singh, Lal Bahadur Singh

**Affiliations:** a Saku Foundation, 142 Prestige Ozone, Whitefield Main Road, Bangalore, 560066, India.; b Department of Medicine, Mahavir Cancer Sansthan, Patna, India.

## Abstract

**Objective:**

To report experiences in Bihar, India’s most densely populated state, with a state government programme to train community health workers (CHWs) to combat the coronavirus disease 2019 (COVID-19) pandemic in the state’s predominantly rural population of 128 million.

**Methods:**

In May 2021, during the second wave of the COVID-19 pandemic in India, the Bihari government initiated a 1-day COVID-19 training programme for rural, unaccredited CHWs who had recently completed a community health education course from the National Institute of Open Schooling. The use of primary health centre buildings and doctors to deliver COVID-19 training and the existence of certification data on CHWs who participated in the community health education course streamlined implementation and minimized costs. After COVID-19 training, CHWs were paid as first responders and COVID-19 treatment workers by the Bihari government.

**Findings:**

Overall, 15 000 CHWs in Bihar completed the COVID-19 training programme in 2021 and a further 30 000 were enrolled. A survey of CHWs carried out after COVID-19 training had started found that 80% (81/102) were satisfied with training and felt they were receiving information from reliable sources.

**Conclusion:**

The training and mobilization of a team of CHWs helped ease pressure on a stressed, rural, health-care system in Bihar and improved its preparedness for future COVID-19 outbreaks. The success of the training programme illustrates how local initiatives can help address gaps in the health workforce and extend the reach of public health care into rural areas, in addition to improving COVID-19 responses.

## Résumé

**Objectif:**

Rendre compte des expériences effectuées au Bihar, l'État le plus densément peuplé d'Inde, dans le cadre d'un programme gouvernemental de formation des agents de santé communautaires (ASC) destiné à lutter contre la pandémie de maladie à coronavirus 2019 (COVID-19) au sein d'une population de 128 millions de personnes, majoritairement issues d'un milieu rural.

**Méthodes:**

En mai 2021, durant la deuxième vague de la pandémie de COVID-19 en Inde, le gouvernement Bihari a instauré un programme de formation à la COVID-19 d'une journée pour les ASC ruraux non agréés qui avaient récemment terminé un cours d'éducation à la santé communautaire au National Institute of Open Schooling. L'utilisation des médecins et des locaux du centre de soins de santé primaires pour assurer la formation à la COVID-19, ainsi que l'existence de données de certification sur les ASC ayant participé au cours d'éducation à la santé communautaire ont facilité la mise en œuvre et limité les coûts. À l'issue de la formation, les ASC étaient rémunérés par le gouvernement Bihari en tant que premiers intervenants et professionnels en matière de prise en charge de la COVID-19.

**Résultats:**

Au total, 15 000 ASC ont suivi le programme de formation à la COVID-19 au Bihar en 2021, et 30 000 autres s'y sont inscrits. Selon une enquête menée auprès des ASC après le lancement de la formation, 80% (81/102) des participants en étaient satisfaits et avaient l'impression de recevoir des informations provenant de sources fiables.

**Conclusion:**

Former et mobiliser une équipe d'ASC a permis de diminuer la pression exercée sur un système de soins de santé rural débordé au Bihar, et de mieux le préparer à de futures épidémies de COVID-19. Le succès de ce programme de formation illustre la manière dont les initiatives locales peuvent contribuer à combler le manque de personnel soignant et rendre les soins de santé publics plus accessibles dans les régions rurales, en plus d'améliorer la lutte contre la COVID-19.

## Resumen

**Objetivo:**

Informar de las experiencias en Bihar, el estado más densamente poblado de la India, sobre la aplicación de un programa del gobierno estatal para formar a los agentes de salud comunitarios (ASC) con el fin de controlar la pandemia de la coronavirosis de 2019 (COVID-19) en una población de 128 millones de personas, en su mayoría de zonas rurales.

**Métodos:**

En mayo de 2021, durante la segunda oleada de la pandemia de la COVID-19 en la India, el gobierno de Bihari puso en marcha un programa de formación sobre la COVID-19 de un día de duración para los ASC de las zonas rurales, sin acreditación, que acababan de terminar un curso de educación de salud comunitaria del Instituto Nacional de Enseñanza Abierta. Gracias a la utilización de los edificios de los centros de salud primaria y de los médicos para impartir la formación sobre la COVID-19 y a la existencia de datos de certificación de los ASC que habían participado en el curso de educación de salud comunitaria, se agilizó la ejecución y se minimizaron los costes. Después de la formación sobre la COVID-19, el gobierno de Bihari pagó a los ASC como personal de primera intervención y atención de la COVID-19.

**Resultados:**

En total, 15 000 ASC de Bihar completaron el programa de formación sobre la COVID-19 en 2021 y otros 30 000 se inscribieron. Una encuesta realizada a los ASC tras el inicio de la formación sobre la COVID-19 reveló que el 80 % (81/102) estaban satisfechos con la formación y opinaban que estaban recibiendo información de fuentes fiables.

**Conclusión:**

La formación y la movilización de un grupo de ASC ayudó a aliviar la presión sobre un sistema sanitario rural sometido a tensión en Bihar y mejoró su preparación para futuros brotes de la COVID-19. El éxito del programa de formación ilustra cómo las iniciativas locales pueden ayudar a subsanar las deficiencias del personal sanitario y ampliar el alcance de la atención sanitaria pública en las zonas rurales, además de mejorar la contención de la COVID-19.

## ملخص

**الغرض:**

إعداد تقارير عن التجارب في ولاية بيهار، الولاية الأكثر كثافة سكانية في الهند، في ظل برنامج حكومي للولاية لتدريب العاملين في مجال الصحة المجتمعية (CHW) لمكافحة جائحة مرض فيروس كورونا 2019 (كوفيد 2019) لدى سكان الولاية الذين يغلب عليهم الطابع الريفي، والبالغ عددهم 128 مليون نسمة.

**الطريقة:**

في مايو/أيار 2021، خلال الموجة الثانية من جائحة كوفيد 19 في الهند، بدأت حكومة بيهار برنامجًا تدريبيًا لمدة يوم واحد حول كوفيد 19 للعاملين في مجال الصحة المجتمعية، من الريفيين غير المعتمدين، الذين أكملوا مؤخرًا دورة التثقيف الصحي المجتمعي من المعهد الوطني للتعليم المفتوح. إن الاعتماد على مباني وأطباء المراكز الصحية الأولية لتقديم تدريب حول كوفيد 19، ووجود بيانات التوثيق الخاصة بالعاملين في مجال الصحة المجتمعية، الذين شاركوا في دورة التثقيف الصحي المجتمعي، أدى جميعه إلى تبسيط التنفيذ والحد من التكاليف. بعد تدريب كوفيد 19، تم دفع رواتب العاملين في مجال الصحة المجتمعية كأول مستجيبين وعاملين في علاج كوفيد 19 من جانب حكومة بيهار.

**النتائج:**

بشكل عام، أكمل 15000 من العاملين في مجال الصحة المجتمعية في ولاية بيهار برنامج تدريب كوفيد 19 في عام 2021، وتم تسجيل 30000 آخرين. تم إجراء مسح للعاملين في مجال الصحة المجتمعية بعد بدء تدريب كوفيد 19، ووجد أن 80% (81/102) كانوا راضين عن التدريب، وشعروا أنهم يتلقون المعلومات من مصادر موثوقة.

**الاستنتاج:**

ساعد تدريب وحشد فريق من العاملين في مجال الصحة المجتمعية في تخفيف الضغط على نظام الرعاية الصحية الريفية المجهد في ولاية بيهار، فضلًا عن تحسين استعداد هذا النظام لحالات تفشي كوفيد 19 في المستقبل. يوضح نجاح برنامج التدريب كيف يمكن للمبادرات المحلية أن تساعد في سد الفجوات في القوى العاملة الصحية، وتوسيع انتشار الرعاية الصحية العامة في المناطق الريفية، بالإضافة إلى تحسين خطوات الاستجابة لكوفيد 19.

## 摘要

**目的:**

旨在报告在印度人口最稠密的比哈尔邦推行邦政府计划的经验，即为社区卫生工作者（以下简称 CHW）提供培训，以应对主要由农村人口（占 1.28 亿）构成的比哈尔邦的新型冠状病毒肺炎（新冠肺炎）疫情。

**方法:**

2021 年 5 月，在印度第二波新型冠状病毒肺炎疫情蔓延期间，面向农村地区未经认证但最近完成了国家开放教育机构提供的社区卫生教育课程的 CHW，比哈尔政府启动了一项为期 1 天的新型冠状病毒肺炎培训计划。通过由初级卫生中心的医生在中心大楼提供新型冠状病毒肺炎相关培训并授予参与社区卫生教育课程的 CHW 证书，该计划简化了实施流程并最大程度地降低了成本。随后，比哈尔政府将接受了新型冠状病毒肺炎相关培训的 CHW 聘请为急救人员和新型冠状病毒肺炎治疗人员并相应地支付报酬。

**结果:**

2021 年比哈尔邦共有 15,000 名 CHW 完成了新型冠状病毒肺炎培训计划，另外还有 30,000 名 CHW 已报名登记。在新型冠状病毒肺炎相关培训启动后面向 CHW 开展的一项调查发现，80% (81/102) 的 CHW 对此类培训感到满意，并认为他们获取的信息非常可靠。

**结论:**

培训和动员 CHW 队伍有助于缓解比哈尔邦不堪重负的农村卫生保健系统的压力，并提高其未来抵抗新一波新型冠状病毒肺炎疫情的能力。培训计划的成功实施表明，除加强新型冠状病毒肺炎应对措施以外，地方政府还能如何采取行动以帮助解决卫生领域的人力缺口问题并将公共卫生保健范围扩大到农村地区。

## Резюме

**Цель:**

Сообщить об опыте работы в штате Бихар, самом густонаселенном штате Индии, с программой Правительства штата по обучению общинных медико-санитарных работников (ОМСР) методам борьбы с пандемией коронавирусной инфекции 2019 года (COVID-19) среди 128-миллионного, преимущественно сельского населения штата.

**Методы:**

В мае 2021 года во время второй волны пандемии COVID-19 в Индии Правительство штата Бихар инициировало однодневную программу обучения по COVID-19 для сельских неаккредитованных ОМСР, которые недавно прошли курс санитарного просвещения на уровне общины в Национальном институте открытого образования. Использование зданий центров первичной медико-санитарной помощи и вовлечение врачей для проведения обучения по COVID-19, а также наличие данных о сертификации ОМСР, участвовавших в курсе санитарного просвещения на уровне общины, упростили реализацию программы и свели расходы к минимуму. После обучения по COVID-19 Правительство штата Бихар платило ОМСР как работникам службы экстренного реагирования, а также как работникам, принимавшим участие в лечении COVID-19.

**Результаты:**

В целом программу обучения по COVID-19 в штате Бихар в 2021 году прошли 15 000 ОМСР и еще 30 000 были включены в нее. Опрос ОМСР, проведенный после обучения по COVID-19, показал, что 80% (81/102) были довольны обучением и считают, что они получили информацию из надежных источников.

**Вывод:**

Обучение и мобилизация команды ОМСР помогли снизить нагрузку на перегруженную сельскую систему здравоохранения в штате Бихар, а также повысили ее готовность к будущим вспышкам COVID-19. Успех программы обучения показывает, как местные инициативы могут помочь устранить пробелы в трудовых ресурсах здравоохранения и расширить сферу охвата общественного здравоохранения в сельских районах, а также улучшить меры реагирования на COVID-19.

## Introduction

Community health workers (CHWs) can extend the reach of health-care systems to low-resource communities.^1^ In India, due to a shortage of doctors,^2^ these health workers play an important role in providing primary health care in rural areas, where 70% of the country’s population resides.^3^ In addition, CHWs played a critical role during the coronavirus disease 2019 (COVID-19) pandemic, particularly in predominantly rural states.^4^

In 2018, the Indian government launched its flagship *Ayushman Bharat* programme (National Health Protection Scheme) to provide universal health care to marginalized communities.^5^ Health and wellness centres were established in villages to deliver basic health services close to rural communities.^6^ In addition, it was hoped that community-centred wellness activities developed through these centres would increase community involvement and lead to better long-term public health outcomes.

Today, health and wellness centres provide preventive, promotive, rehabilitative and curative care for an expanded range of services encompassing reproductive and child health, communicable and noncommunicable diseases, palliative and elderly care, basic emergency care, oral health and ear, nose and throat care.^7^ Each centre typically serves 10 000 people and is supported by a trained primary health care team of nonphysician health workers, which often comprises two trained multipurpose workers (male or female educated to grade 12, including science), five government-accredited social health activist workers who perform outreach, and one mid-level health provider (typically a nurse or someone with a degree in community health).^8^ Accredited social health activist workers are female village residents who act as CHWs.^9^^,^^10^ Their role was created in 2005 to provide maternal and newborn health care; however, over the years their work has expanded to cover other health campaigns that require outreach, as well as domestic and community-based services. Clusters of health and wellness centres are linked to primary health centres that provide more extensive medical services for populations of around 50 000 and that serve as the first points of referral and as administrative hubs.^11^ Each primary health centre is staffed by at least one qualified doctor, one staff nurse, one pharmacist and one laboratory technician. In November 2020, more than 50 000 health and wellness centres were operational across India.^12^ The goal is to establish a network of 150 000 centres by December 2022.^13^

Besides the accredited social health activist workers who provide frontline services in communities as part of the *Ayushman Bharat* initiative, there is also a large number of unaccredited CHWs who are self-trained and who have picked up basic skills working, for example, in nursing homes or pharmacies. Local communities with whom CHWs have built a relationship of trust regard them as doctors and approach them as the first points of contact for primary health care. These unaccredited CHWs played a critical role in responding to the COVID-19 pandemic as it swept through the country, especially in predominantly rural states like Bihar.^14^ Bihar is India’s most densely populated state, is the third largest state by population and 89% of its 128 million inhabitants live in rural areas.^15^ Although unaccredited CHWs see a large number of people, they do not have formal training and may, therefore, pose a risk to patients. The aim of this paper was to report the results of a recent initiative by the Bihari government to train unaccredited CHWs, particularly in response to the COVID-19 pandemic, and to bring them into the mainstream health-care system. A similar model could be adopted by other states and developing nations that are struggling with a shortage of frontline health-care workers, especially in rural areas.

## Methods

The first case of COVID-19 was reported in India in January 2020 and the infection rate was under control by February 2021. However, the number of cases started trending upwards again in March 2021, which led to a second wave of infection. By May 2021, more than 400 000 new cases were being reported each day. According to the Centre for Science and Environment in India, this wave was due primarily to a surge in cases in rural districts.^16^

**Fig. 1:**
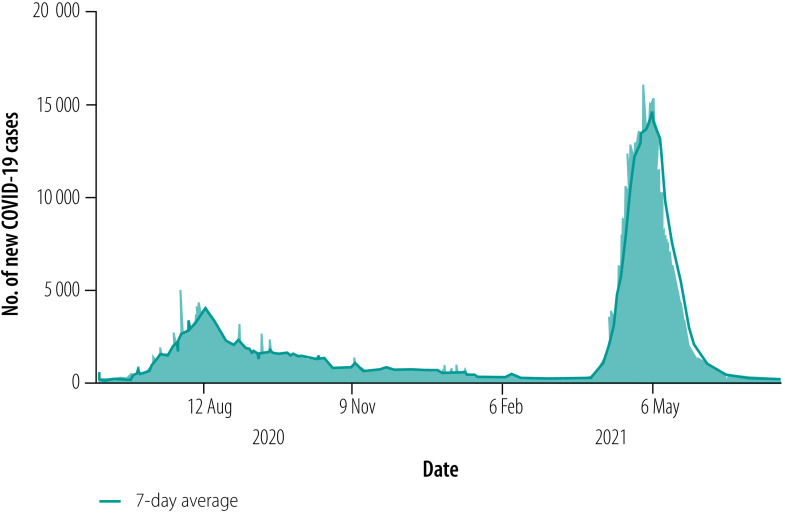
Number of new COVID-19 cases per day, Bihar, India, 2020–2021

The state of Bihar reported its first COVID-19 case in March 2020. During the first wave, the state witnessed a surge driven by migrant workers returning home from different parts of India following an abrupt national lockdown. In March 2021, Bihar was hit by an enormous second wave, with rural areas affected on a larger scale. The primary driver was thought to be the influx of migrant workers from other states, which coincided with a major holiday. At the peak in May 2021, more than 15 000 new cases were reported each day in Bihar (Fig. 1). Moreover, health experts believe that the number of cases was substantially underreported because of a lack of adequate testing infrastructure in rural areas.^18^ The second wave exposed weaknesses in the rural health-care infrastructure and the lack of trained health-care workers. The Centre for Science and Environment has estimated that in 2021 rural areas in India needed 76% more doctors to manage primary health care.^16^

COVID-19: coronavirus disease 2019.

Notes: The second wave of the coronavirus disease 2019 (COVID-19) pandemic in Bihar peaked on 6 May 2021, when there were 15 126 new cases reported and the 7-day average was 14 191 cases per day. Data were obtained from an online interactive COVID-19 dashboard hosted by Johns Hopkins University.^17^

### Community health worker training

In May and June 2021, the Bihari government implemented a specialized training programme designed to enable the large workforce of unaccredited CHWs to help manage the COVID-19 outbreak at the grassroots level. The State Health Society of the Bihar Department of Health estimated that there were more than 400 000 CHWs in the state in 2015.^19^ An initiative to educate CHWs about community health had already started before the pandemic in September 2019. The approach to COVID-19 adopted was to supplement the community health education initiative with focused COVID-19 training and to establish a formal structure to enlist the help of CHWs in managing COVID-19. 

To train unaccredited CHWs in community health, the State Health Society signed a memorandum of understanding with the National Institute of Open Schooling, which is an autonomous organization set up by the Indian Ministry of Education. In addition, the Society constituted a nine-member advisory committee to monitor the training course, which was named *Jan Swasthya Pathyakram* (i.e. community health education). The National Institute of Open Schooling in partnership with the State Health Society designed the curriculum, which covered general health, a balanced diet, malnutrition, mother and child care, and family welfare. The course ran for over 400 hours and provided both theoretical and practical modules which included self-learning material, audiovisual programmes and hands-on training.^20^ Training was carried out at all 397 functioning primary health centres across the 38 districts of Bihar. Participants were enrolled through newspaper advertisements and social media.^21^ Eligible candidates had to have successfully completed grade 10 of schooling and to have worked as a CHW for 3 to 5 years. The fee was 5000 Indian rupees (₹; equivalent to around 70 United States dollars; US$), which covered the costs of the study materials and the 1-year training course. Participants were registered with the Patna branch of the National Institute of Open Schooling and training was conducted by qualified doctors attached to primary health centres. At the end of training, participants had the option to take a CHW certification examination, which comprised theoretical and experiential modules, at an additional cost of 900 ₹ (approximately US$ 12) at central government schools (i.e. *Kendriya Vidyalayas*). On passing the examination, participants received a course completion certificate from the National Institute of Open Schooling, which was evidence that the health worker was eligible for government employment. Each primary health centre maintained contact with, and aided, health workers trained at their facility. By May 2021, over 15 000 CHWs had completed the community health education training course and passed the certification examination. Subsequently, an estimated 30 000 CHWs were either undergoing training or waiting to take the examination.

### COVID-19 training

At the onset of the second wave of the COVID-19 pandemic in March 2021, the absence of a medical workforce in rural areas created a logistical plight for the Indian government.^22^ However, even without special training, CHWs were helping their local communities by encouraging the behavioural changes needed to prevent infection, such as social distancing, wearing face masks, hygiene measures and adoption of the *Aarogya Setu* mobile phone application developed by the Indian government, which provided people with information about individual and local risks of infection.^23^ The government recognized the importance of officially deploying the services of CHWs and, in May 2021, the Bihar Department of Health produced a document on the induction of CHWs into formal roles after they had undergone specific COVID-19 training. Here, we report this new development in accordance with the United Nations Children's Fund’s framework for optimizing community health programmes: the Community Health Worker Assessment and Improvement Matrix.^24^

The first step was to provide formal training on responses to the COVID-19 pandemic to the first 15 000 rural CHWs who had completed the community health education training course and passed the certification examination. All primary health centres in the state of Bihar were instructed to contact CHWs who had undergone training and to ask them to report back to their centres for COVID-19 training. Thereafter, the CHWs were enlisted and paid by the state government as first responders and COVID-19 treatment support workers.

A 1-day COVID-19 training programme was designed to enable CHWs: (i) to safely identify potential cases of COVID-19 based on symptoms, while maintaining social distancing; (ii) to liaise with the nearest primary health centre to arrange testing and treatment; (iii) to monitor patients confirmed as having COVID-19 who were isolating at home; (iv) to coordinate activities with the local district control centre; (v) to refer patients with serious symptoms to dedicated COVID-19 health centres in the district; and (vi) to maintain a list of all patients seen by CHWs. Training was carried out by doctors in primary health centres with groups of 70 to 80 CHWs. 

Even after training, CHWs did not carry out polymerase chain reaction testing (PCR). Instead, they were asked to refer patients for testing at primary health centres. In general, symptomatic COVID-19 patients were referred to primary health centres that had test facilities and links to dedicated COVID-19 health centres. However, CHWs were authorized to prescribe the standard government recommended list of COVID-19 medications. Primary health centres assisted CHWs on an ongoing basis with any help required. Although the Bihari government did not provide any special protective equipment as part of COVID-19 training, basic supplies, such as masks, gloves and soap, were distributed at the community level. In addition, nongovernmental organizations provided funding for, and distributed, protective kits to CHWs.

The State Health Society in Bihar was responsible for the overall monitoring and supervision of the CHW training programme in collaboration with the central National Institute of Open Schooling and primary health centres across the state. In addition, primary health centres were responsible for monitoring the quality of their own training and, if required, submitted feedback on areas of improvement to district health officers. Data on CHW training were collected by each primary health centre and reported to the State Health Society and the National Institute of Open Schooling. The data were used to improve the coverage and quality of the training programme and to help plan mass community campaigns.

No additional examination or certification was offered to CHWs after completion of the COVID-19 training because the COVID-19 module was made available only to workers who had already completed the community health education training course and passed the certification examination. Nevertheless, the Bihari government regards these CHWs as capable of spearheading community campaigns, such as the campaign against malnutrition held on village health and nutrition days and community awareness campaigns aimed at preventing communicable diseases such as tuberculosis. After completing COVID-19 training, CHWs participated in community actions against COVID-19, such as vaccination campaigns. Although these CHWs are not salaried, there are financial incentives. The Bihari government decided to award CHWs a fixed payment of 200 ₹ (approximately US$ 3) for each COVID-19 patient they handled and referred to primary health centres. The CHWs had to submit patient details (i.e. a mobile phone number and a unique national identification number) to the relevant centre to be eligible for the payment.^25^

Practising CHWs are usually members of a *Graameen Chikitsa Seva Samanvay Samiti* (rural medical services coordination committee), which is a key community forum for facilitating information sharing and which acts as a peer support group. These committees, which operate at village and district levels, promote the use of accurate information, conduct sessions with experts and encourage health workers with experience in hospitals, nursing homes or clinics to participate in their local communities.

### Funding

Health and wellness centres and primary health centres are operating under the *Ayushman Bharat* programme and the budget for these facilities is primarily covered by central government, with some support from state governments. Although the programme funds the training of accredited social health activist workers, it does not cover the funding for training unaccredited CHWs. In Bihar, CHW training undertaken by the state government was primarily funded by the registration fees for the National Institute of Open Schooling’s community health education training programme. The small additional funding required to create the add-on COVID-19 training module was provided by the State Health Society of the Government of Bihar.

## Results

A survey of 547 CHWs in Bihar carried out in April 2021 before the Bihari government decided to formally enlist them in the COVID-19 response found that 88% (481/547) thought that people in the community used them as the first points of contact for primary health care and 75% (410/547) stated they had not received any COVID-19 training or information directly from the government. Instead the CHWs had obtained basic information about handling COVID-19 cases though informal channels such as social media. Despite the lack of formal training, CHWs carried out their regular duties (exposing themselves and their families to the risk of infection) and their work hours increased substantially.

Certification in community health education by the National Institute of Open Schooling created a potential livelihood for CHWs. They became eligible to deliver an expanded range of national and state health services on behalf of the government, such as: (i) awareness campaigns about epidemics and communicable diseases like tuberculosis; (ii) prevention, screening and referral for noncommunicable diseases such as diabetes; and (iii) screening for basic mental health, oral, and ear, nose and throat conditions. Moreover, the introduction of COVID-19 training and remuneration on a per-case basis not only supplemented their income, but also better equipped them to handle COVID-19 outbreaks in the community. In addition, the participation of CHWs helped ease the strain on frontline medical workers who were struggling to cope with multiple COVID-19 outbreaks. More broadly, the availability of trained personnel ready to take part in different projects augmented the capacity of the public health-care infrastructure and brought services closer to the community.

**Fig. 2:**
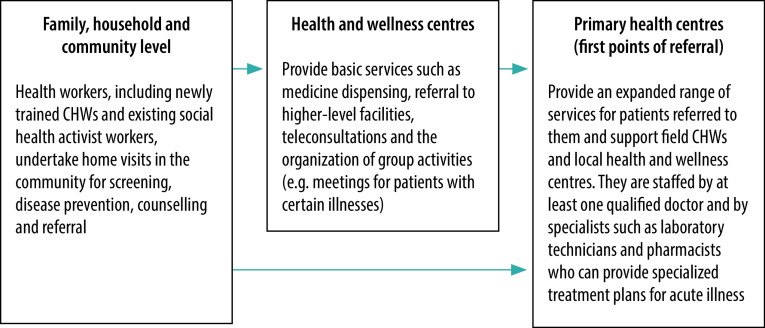
Role of newly trained community health workers in the national health system, Bihar, India, 2021

A survey of 102 CHWs in the districts of Patna and Vaishali in Bihar carried out in June 2021 after the introduction of COVID-19 training found that 80% (81/102) were satisfied that their training needs were being met and that they were receiving information from reliable sources. In addition, a survey of 16 primary health centres in Patna and 11 primary health centres in Vaishali carried out in June 2021 reported that the average number of people with COVID-19 symptoms referred to each centre by CHWs increased from 5 per day before COVID-19 training to 15 per day after. To cater for the increased number of referrals from rural districts, the Bihari government expanded the capacity of dedicated COVID-19 isolation and treatment centres in each district.^26^ Once CHWs were incorporated into the mainstream health system, they were able to help deliver services in rural areas through multiple channels, as illustrated in Fig. 2.

CHW: community health worker.

Notes: Health and wellness centres are located in villages and deliver basic health services close to rural communities. Each primary health centre is linked to a cluster of health and wellness centres.

## Discussion

The mobilization of rural CHWs by the Bihari government during the second wave of the COVID-19 pandemic helped ease the pressure on a stressed health-care system and improved its preparedness for future outbreaks. However, the process of inducting and training CHWs and implementing safety measures for them could have started earlier after the first COVID-19 wave. By the time infections spiked in rural areas during the second wave, many CHWs had already reduced their activities and scaled back door-to-door visits due to a lack of information, training and protective equipment.

Creating a training structure for CHWs across the whole of Bihar was complicated by: (i) the geographical spread of CHWs; (ii) the need to exchange information with a large number of health workers; and (iii) the limited availability of trainers and training venues. The Bihari government tackled these logistical issues and quickly delivered the COVID-19 training programme by taking advantage of the *Ayushman Bharat’s* primary health centre infrastructure and the community health education certification provided by the National Institute of Open Schooling. In particular, the use of primary health centre buildings and doctors to deliver training (both in person and virtually) and the existence of National Institute of Open Schooling certification data on CHWs trained at primary health centres were crucial for streamlining the implementation of COVID-19 training without adding overhead costs.

The Bihari government intends to approve a proposal to merge the COVID-19 training module with the National Institute of Open Schooling’s community health education certification programme for future participants. This change would ensure that the expansion of the COVID-19 training programme is sustainable because more health workers will be able to enrol and gain certification. Moreover, extra costs would be minimal for government because the certification programme is primarily funded by registration fees for the course and examination and because training will make use of existing primary health centre resources (i.e. doctors and venues).

Integrating the training given by central government to accredited social health activist workers with the training given by state governments to unaccredited CHWs would create parity in the training and remuneration received by these two categories of health worker. As the demarcation between accredited social health activist workers and other CHWs becomes more blurred, a larger pool of health workers with common training and incentives could be created. Additionally, once they have received training through the Bihari government, CHWs could aspire to secure salaried jobs in health and wellness centres established by the Indian government in its *Ayushman Bharat* programme. In places like Bihar, where there is a large rural population and limited availability of doctors, it is essential that CHWs receive training and are integrated into the existing health-care system where they can help increase the reach of public health care and contribute to the fight against future waves of the COVID-19 pandemic.
